# Mild therapeutic hypothermia shortens intensive care unit stay of survivors after out-of-hospital cardiac arrest compared to historical controls

**DOI:** 10.1186/cc6925

**Published:** 2008-06-14

**Authors:** Christian Storm, Ingo Steffen, Joerg C Schefold, Anne Krueger, Michael Oppert, Achim Jörres, Dietrich Hasper

**Affiliations:** 1 Charité Universitätsmedizin Berlin, Campus Virchow-Klinikum, Department of Nephrology and Medical Intensive Care Medicine, Augustenburger Platz 1, 13353 Berlin, Germany

## Abstract

**Introduction:**

Persistent coma is a common finding after cardiac arrest and has profound ethical and economic implications. Evidence suggests that therapeutic hypothermia improves neurological outcome in these patients. In this analysis, we investigate whether therapeutic hypothermia influences the length of intensive care unit (ICU) stay and ventilator time in patients surviving out-of-hospital cardiac arrest.

**Methods:**

A prospective observational study with historical controls was conducted at our medical ICU. Fifty-two consecutive patients (median age 62.6 years, 43 males, 34 ventricular fibrillation) submitted to therapeutic hypothermia after out-of-hospital cardiac arrest were included. They were compared with a historical cohort (n = 74, median age 63.8 years, 53 males, 43 ventricular fibrillation) treated in the era prior to hypothermia treatment. All patients received the same standard of care. Neurological outcome was assessed using the Pittsburgh cerebral performance category (CPC) score. Univariate analyses and multiple regression models were used.

**Results:**

In survivors, therapeutic hypothermia and baseline disease severity (Acute Physiology and Chronic Health Evaluation II [APACHE II] score) were both found to significantly influence ICU stay and ventilator time (all *P *< 0.01). ICU stay was shorter in survivors receiving therapeutic hypothermia (median 14 days [interquartile range (IQR) 8 to 26] versus 21 days [IQR 15 to 30] in the control group; *P *= 0.017). ICU length of stay and time on ventilator were prolonged in patients with CPC 3 or 4 compared with patients with CPC 1 or 2 (*P *= 0.003 and *P *= 0.034, respectively). Kaplan-Meier analysis showed improved probability for 1-year survival in the hypothermia group compared with the controls (log-rank test *P *= 0.013).

**Conclusion:**

Therapeutic hypothermia was found to significantly shorten ICU stay and time of mechanical ventilation in survivors after out-of-hospital cardiac arrest. Moreover, profound improvements in both neurological outcome and 1-year survival were observed.

## Introduction

Persistent coma is a common finding after cardiac arrest and has profound ethical and economic implications. In a significant proportion of patients, neurological status rather than specific treatment of the underlying disease affects the outcome after cardiac arrest [1]. Recent randomized controlled trials have demonstrated that therapeutic hypothermia is highly effective in improving the neurological outcome in patients after cardiac arrest [2,3]. In 2003, the International Liaison Committee on Resuscitation (ILCOR) recommended this treatment for all comatose survivors of out-of-hospital cardiac arrest due to ventricular fibrillation [4]. Although only a minority of such patients are currently treated with therapeutic hypothermia [5,6], recent efforts aim to implement therapeutic hypothermia as a routine procedure in patients after cardiac arrest [7].

The optimal method for controlled and safe application of therapeutic hypothermia is still under debate [8,9]. For intravascular cooling devices as well as for device-controlled surface cooling methods, the efficacy has been demonstrated in different trials [10-13]. Of course, other cooling methods like crushed-ice, towels pre-soaked in ice water, or simple cooling blankets may be very effective as well but the temperature range is more difficult to control [14,15]. For out-of-hospital cooling, new devices and methods for fast induction of hypothermia are of increasing importance [16,17]. However, using advanced cooling methods, equipment, and manpower required for the application of therapeutic hypothermia generates higher treatment costs. Length of intensive care unit stay (ICU LOS) has been identified as a major determinant of total treatment costs after survived cardiac arrest [18]. Therefore, it was investigated whether therapeutic hypothermia influences ICU LOS and time of mechanical ventilation in patients after out-of-hospital cardiac arrest.

## Materials and methods

The study protocol was approved by the local ethics committee on human research. The need for informed patient consent was waived by the committee. We conducted our study in an urban area with a two-tiered medical emergency system: basic life support, including automated defibrillation, is offered by ambulances, and advanced life support procedures were performed by qualified emergency physicians at the mobile ICU. All patients with cardiac arrest and return of spontaneous circulation (ROSC) in the field were directly admitted to our medical ICU (MICU). Pre-hospital cooling procedures were not applied during the study period.

Between January 2006 and January 2007, a total of 52 patients were admitted to our MICU after out-of-hospital cardiac arrest. All patients received therapeutic hypothermia according to the current ILCOR recommendations. A historical control group in the era prior to hypothermia treatment was identified in a cohort of 74 patients admitted to our MICU between 2003 and 2005 after out-of-hospital cardiac arrest. Detailed characteristics for all study patients are presented in Table 1.

**Table 1:** Baseline characteristics of the patient population and results of the univariate analysis

	Control	Hypothermia	*P *value
	(n = 74)	(n = 52)	
Age, years	63.8 (52.8–72.0)	62.6 (50.7–71.4)	0.776
Gender			
Female	21 (28.4)	9 (17.3)	0.221
Male	53 (71.6)	43 (82.7)	
APACHE II score	25.0 (20.0–30.0)	30.5 (22.5–33.0)	0.015
Cardiac arrest			
Shockable rhythm	43 (58.1)	34 (65.4)	0.523
Non-shockable rhythm	31 (41.9)	18 (34.6)	
Cause of cardiac arrest			
Acute myocardial infarction	47 (63.5)	31 (59.6)	
Primary arrhythmia	12 (16.2)	13 (25)	
Respiratory	12 (16.2)	8 (15.4)	
Other	3 (4.1)	0	
Bystander CPR			
Yes	12 (16.2)	16 (30.8)	0.086
No	62 (83.8)	36 (69.2)	
Time to ROSC, minutes	20 (18–25)	20 (14–22)	0.230
Total epinephrine dose, mg	3.0 (2.0–4.0)	2.75 (0.8–3.1)	0.106
Length of ICU stay, days			
All patients	15 (7–26)	13 (8–26)	0.947
Non-survivors	7 (5–10)	9 (6–22)	0.259
Survivors	21 (15–30)	14 (8–26)	0.017
Time on ventilator, hours			
All patients	217 (139–353)	220 (124–428)	1
Non-survivors	165 (104–210)	221 (133–365)	0.219
Survivors	328 (208–461)	219 (125–447)	0.113
Leukocytes at day 3,/nL	11.0 (8.7–13.6)	12.2 (8.3–14.1)	0.91
C-reactive protein at day 3, mg/dL	12.7 (7.5–16.0)	14.5 (8.7–21.1)	0.33
Radiological signs of pneumonia	8 (10.8)	10 (19.2)	0.205
CPC at ICU discharge			
1 – good recovery	8 (10.8)	22 (42.3)	
2 – moderate disability	9 (12.2)	10 (19.2)	
3 – severe disability	3 (4.1)	2 (3.8)	
4 – vegetative state	23 (31.1)	3 (5.8)	
5 – death	31 (41.9)	15 (28.8)	
CPC 1–2 versus 3–5			
CPC 1–2	17 (23.0)	32 (61.5)	<0.001
CPC 3–5	57 (77.0)	20 (38.5)	
Died during hospital stay			
Yes	31 (41.9)	15 (28.8)	0.190
No	43 (58.1)	37 (71.2)	

Data are presented as medians (25th and 75th percentiles) or as absolute numbers (relative frequencies). APACHE II, Acute Physiology and Chronic Health Evaluation II; CPC, cerebral performance category; CPR, cardiopulmonary resuscitation; ICU, intensive care unit; ROSC, return of spontaneous circulation.

Therapeutic hypothermia was initiated after admission with an intravenous infusion of cold saline (4°C, 1,000 to 1,500 mL bolus) followed by surface cooling with commercially available non-invasive devices (CritiCool^®^; MTRE, Yavne, Rehovot, Israel, or ArcticSun2000^®^; Medivance, Inc., Louisville, CO, USA). After the start of cooling, the target temperature of 33°C was reached in a range of 180 to 300 minutes and maintained for 24 hours. Re-warming was performed at a rate of 0.25°C per hour. Intravenous sedation was induced in all patients by a combination of midazolam (0.125 mg/kg per hour) and fentanyl (0.002 mg/kg per hour) with dose adjustment as needed. Patients undergoing hypothermia received muscle relaxation with repetitive pancuronium (0.1 mg/kg) administration in order to prevent shivering. All patients completed therapeutic hypothermia without complications or overcooling. With the exception of therapeutic hypothermia, there was no further difference in the standard of intensive care treatment between the two groups. To discover early-onset infection and pneumonia, laboratory markers of systemic inflammation (c-reactive protein/leukocyte count) and radiological findings of pneumonia in chest x-ray were analyzed on day 3. Neurological outcome was assessed at the time of discharge from the ICU according to the Pittsburgh cerebral performance category (CPC) score [19]. CPC 1 and 2 were classified as a favorable neurological outcome, whereas CPC 3 and 4 were regarded as an unfavorable outcome.

The SPSS software (version 13.0; SPSS Inc., Chicago, IL, USA) and R (version 2.4.1; The R Foundation for Statistical Computing) were used for statistical analysis. Descriptive parameters are presented as median and interquartile range (IQR) (25th to 75th percentiles). Univariate analysis of differences between hypothermia patients and the control group was performed by using the Mann-Whitney *U *test for non-parametric unpaired data and the Fisher exact test for dichotomous variables. Multiple regression analysis was used to adjust for confounders and to analyze the association of one dependent and several independent variables. The general linear start model includes all independent variables, whereas the final model contains only predictive factors after using the automatic method of stepwise backward selection. Survival data were analyzed by the Kaplan-Meier method, and comparison between groups was performed by the log-rank test.

## Results

### Univariate analysis

Results of univariate analysis of all patients (n = 126) are presented in Table 1. Concerning age (*P *= 0.776) and gender (*P *= 0.221), no significant differences were observed between patients in the hypothermia group and the control group. However, APACHE II (Acute Physiology and Chronic Health Evaluation II) score on admission was significantly lower in the control group (*P *= 0.015). No significant differences were calculated concerning the first documented rhythm (*P *= 0.523), dosage of epinephrine during resuscitation (*P *= 0.106), and time to ROSC (*P *= 0.230). Patients with initial ventricular fibrillation were classified as primary shockable rhythm, whereas patients with asystole and pulseless electrical activity were classified as primary non-shockable rhythm.

**Figure 1:**
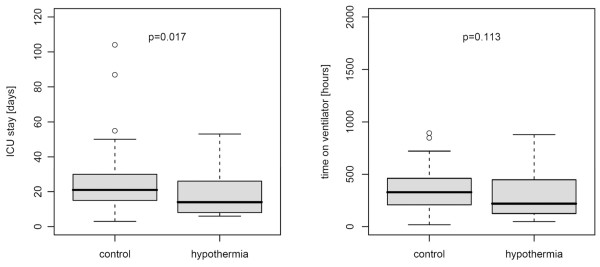
Intensive care unit (ICU) length of stay and time on ventilator in the study groups. Boxplot of ICU length of stay (left) and time on ventilator (right) in survivors of the hypothermia (n = 23) and the control (n = 43) group.

Univariate analysis of ICU LOS for all patients (dead and alive) showed no significant difference between the two groups (*P *= 0.947). However, if only survivors were analyzed, ICU LOS was significantly lower in the hypothermia group (median 14 days [IQR 8 to 26] versus 21 days [IQR 15 to 30]; *P *= 0.017) (Figure 1). In contrast to this finding, there was no statistically significant difference regarding ICU LOS in patients who died during ICU stay (median 9 days [IQR 6 to 22] for the hypothermia group and 7 days [IQR 5 to 10] for the control group; *P *= 0.250).

Univariate analysis of ventilator time revealed no significant difference for all patients (*P *= 1). Median ventilator time was lower in the subgroup of survivors treated with hypothermia, although the difference was not statistically significant (219 hours [IQR 125 to 447] for the hypothermia group versus 328 hours [IQR 208 to 461] for the control group; *P *= 0.113) (Figure 1). Also, there was no statistically significant difference in ventilator time in the subgroup of non-survivors (221 hours [IQR 133 to 365] for the hypothermia group and 165 hours [IQR 104 to 210] for the control group; *P *= 0.219).

**Figure 2:**
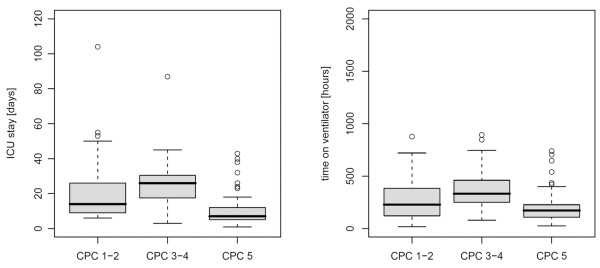
Intensive care unit (ICU) length of stay and time on ventilator and neurological outcome. Boxplot of ICU length of stay (left) and time on ventilator (right) of the study population (n = 126) according to the neurological outcome assessed as cerebral performance category (CPC).

C-reactive protein, leukocyte count, and radiological diagnosis of early-onset pneumonia on day 3 were not statistically different between the groups (Table 1). With regard to the neurological outcome, both ICU LOS and time on ventilator were significantly longer in patients with a CPC 3 or 4 compared with patients with CPC 1 or 2 (*P *= 0.003 for LOS and *P *= 0.034 for ventilator time) (Figure 2). Hypothermia treatment was associated with significantly improved neurological outcome assessed by CPC 1 or 2 versus 3 or 4 (*P *< 0.001). The results of the univariate analysis are summarized in Table 1.

### Multivariate analysis

As univariate analysis showed significant differences between the hypothermia and the control group, an adjustment for confounders was performed. To this end, ICU LOS and respirator time were analyzed with the multivariate regression model, including gender, age, APACHE II score, bystander cardiopulmonary resuscitation (CPR), time to ROSC, initial rhythm, and mild therapeutic hypothermia treatment as independent factors. The final model for ICU LOS in the subgroup of survivors identified a low APACHE II score (*P *= 0.006) and hypothermia treatment (*P *= 0.004) as independent predictors of shorter ICU LOS.

**Figure 3:**
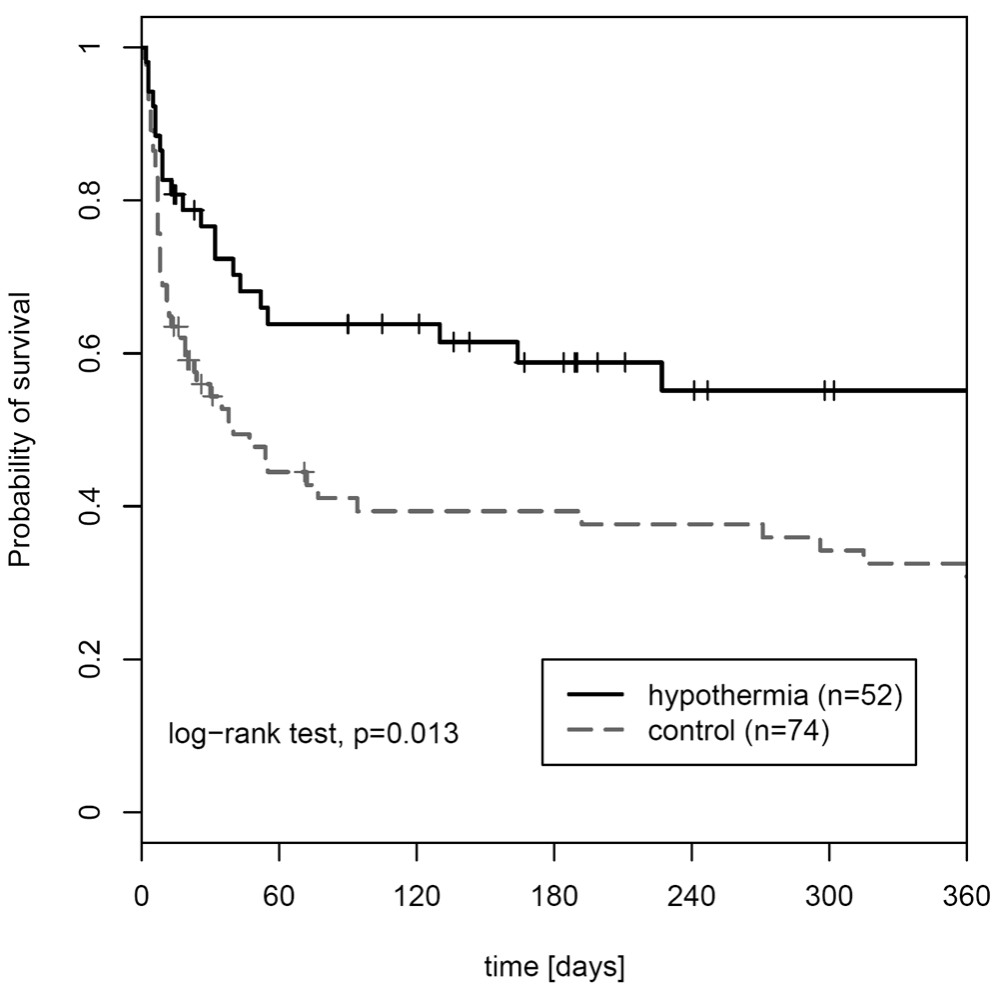
One-year survival of the study population. Kaplan-Meier 1-year survival analysis of both study groups.

Likewise, multivariate analysis in the subgroup of survivors identified APACHE II score (*P *= 0.009) and hypothermia treatment (*P *= 0.026) as independent predictors of shorter time on mechanical ventilation. The complete results of the multivariate analyses are presented in Table 2. Follow-up data for 1-year survival probability were performed including all patients (n = 126). Kaplan-Meier analysis revealed a probability for 365-day survival of 55.1% (confidence interval [CI] 39.1% to 68.5%) in the hypothermia group compared with 30.8% (CI 19.9% to 42.3%) in the control group. The log-rank test was significant (*P *= 0.013) (Figure 3).

**Table 2:** Multivariate analysis

	Coefficient	5% CI	95% CI	*P *value
Start model (ICU LOS)				
(Intercept)	7.84	-17.32	33.01	0.543
Gender female	-4.72	-14.12	4.67	0.328
Age	0.08	-0.19	0.36	0.553
APACHE II score	0.66	0.18	1.13	0.008
Hypothermia treatment	-10.63	-17.93	-3.32	0.006
Bystander CPR	-1.69	-9.57	6.20	0.676
Ventricular fibrillation	0.95	-6.71	8.61	0.809
Time to ROSC	-0.12	-0.73	0.49	0.706
Final model (ICU LOS)				
(Intercept)	10.80	-0.64	22.24	0.068
APACHE II score	0.63	0.19	1.07	0.006
Hypothermia treatment	-10.66	-17.73	-3.59	0.004
Start model (time on ventilator)				
(Intercept)	161.45	-409.67	732.58	0.581
Gender female	2.11	-212.22	216.44	0.985
Age	0.67	-5.40	6.74	0.830
APACHE II score	13.40	3.07	23.74	0.013
Hypothermia treatment	-182.72	-342.75	-22.69	0.028
Bystander CPR	-73.32	-245.39	98.75	0.407
Time to ROSC	-5.16	-19.03	8.71	0.469
Ventricular fibrillation	22.19	-145.07	189.46	0.780
Final model (time on ventilator)				
(Intercept)	107.25	-142.68	357.17	0.403
APACHE II score	13.07	3.58	22.57	0.009
Hypothermia treatment	-179.41	-333.73	-25.09	0.026

Regression coefficients, associated confidence intervals, and *P *values of start multiple regression model and final model after stepwise backward selection. Sign (- or +) indicates negative or positive effect on the dependent variable. APACHE II, Acute Physiology and Chronic Health Evaluation II; CI, confidence interval; CPR, cardiopulmonary resuscitation; ICU LOS, intensive care unit length of stay; ROSC, return of spontaneous circulation.

## Discussion

Mild therapeutic hypothermia is recommended by current ILCOR guidelines and has become an essential part in post-resuscitation care. Although the benefit concerning neurological outcome could not be demonstrated in a few single centers, most other trials could show an improved neurological outcome under hypothermia even in unselected effectiveness trials [20,21]. Nevertheless, technical, logistical, and financial barriers may limit the transfer into daily practice [22]. Therefore, for the first time, the present study investigates the impact of therapeutic hypothermia on ICU LOS in patients with out-of-hospital cardiac arrest. The major determinants of short ICU LOS and ventilator time in patients after cardiac arrest were found to be either early death during ICU stay or rapid neurological recovery. While comparison of all patients (survivors and non-survivors) did not reveal a statistical difference regarding ICU LOS, subgroup analysis showed that short ICU LOS is related to an improved neurological outcome in the hypothermia group. In contrast, short ICU LOS in the control group was associated with a high rate of non-survivors. This striking result was confirmed with a multivariate regression model emphasizing the impact of therapeutic hypothermia on ICU LOS.

A high APACHE II score at admission was associated with a prolonged ICU stay in our study. It seems plausible that patients with higher severity of illness depend more on critical care facilities; however, it is known that the predictive value of the APACHE II score has various limitations after cardiac arrest [23,24]. Our present results suggest that prognostic application of current scoring systems may be even more difficult in patients treated with therapeutic hypothermia. Both ICU LOS and time of mechanical ventilation tended to be longer in non-survivors of the hypothermia group compared with non-survivors of the control group, which may reflect the difficulty of outcome prediction in patients routinely requiring sedation and muscle relaxation in the first days of intensive care. Beside clinical evaluation, biochemical markers (neuron-specific enolase, protein S100B) and electrophysiological studies (somatosensory-evoked potentials) are established tools for outcome prediction in patients after cardiac arrest [25,26]. However, most of these parameters were studied in patients not undergoing hypothermia treatment. At present, little data on whether therapeutic hypothermia treatment may influence these biomarkers are available [27,28]. Therefore, physicians should be careful in the prognostication of patients treated with therapeutic hypothermia [29].

It should be mentioned that our analysis has various limitations. First of all, results obtained with an observational study design using a historical control group generally require further validation by a randomized controlled trial. However, ethical concerns will most likely prevent a further randomized trial that withholds hypothermia treatment from a control group. Second, our local treatment standards may have affected ICU LOS and patient outcome. There is considerable variation regarding end-of-life decisions and care practices. Thus, the approach used by a specific ICU on deciding whether to withhold or withdraw critical care in patients after cardiac arrest may have an important impact on ICU LOS. This may help to explain data from a Canadian survey reporting that patients with a higher Glasgow Coma Scale score had a longer ICU stay [30].

Moreover, differences in ICU LOS may also be related to a different use and allocation of ICU capacities in general. Decision making potentially may be influenced by a variety of factors that are independent of the individual patient characteristics, such as the availability of ICU beds and/or of facilities of 'step-down' care. It is of interest to note, however, that the necessity of mechanical ventilation, which is a rather objective criterion for the necessity of ICU treatment, is reduced by hypothermia treatment. Since impaired neurological status has been identified as a predictor of extubation failure or weaning failure [31,32], this finding may be associated with the more favorable neurological outcome in the hypothermia group.

Another issue with impact on ICU LOS and time on ventilator might be systemic inflammation after cardiac arrest. Both infectious and non-infectious systemic inflammations are a frequent problem in these patients [33,34]. As demonstrated in the HACA (Hypothermia after Cardiac Arrest) trial, there may even be a trend toward a higher rate of infections in patients treated with therapeutic hypothermia. However, we found no difference between the two study groups with respect to laboratory markers of inflammation (C-reactive protein/leukocyte count) and radiological findings of early-onset pneumonia. Therefore, early infection or pneumonia had no impact on different mechanical ventilator times between the groups.

In our analysis, we may have suffered from a selection bias because some baseline characteristics (bystander CPR and rate of ventricular fibrillation) tended to be more favorable in the hypothermia group although APACHE II score was lower in the control group. Finally, we cannot exclude the fact that the introduction of therapeutic hypothermia itself has focused the attention of the physicians in charge to a more sophisticated post-resuscitation care in general, which has been described [35]. However, similar promising results were also reported from a large hypothermia registry [36].

## Conclusion

In summary, patients treated with therapeutic hypothermia showed both an impressive improvement of neurological outcome as well as an increased 1-year survival rate. Furthermore, therapeutic hypothermia did not prolong ICU stay or time of mechanical ventilation; rather, these parameters were reduced in survivors when therapeutic hypothermia was applied. Although we did not directly calculate the ICU treatment costs, we believe that this could be an additional argument for the application of therapeutic hypothermia in patients after cardiac arrest.

## Key messages

• The major determinants of short intensive care unit (ICU) length of stay and ventilator time in patients after cardiac arrest were found to be either early death during ICU stay or rapid neurological recovery.

• Therapeutic hypothermia did not prolong ICU stay or time of mechanical ventilation in patients after cardiac arrest compared with historical controls.

• Both ICU stay and time of mechanical ventilation were reduced in survivors of cardiac arrest treated with therapeutic hypothermia.

• Therapeutic hypothermia was associated with significantly improved neurological outcome and 1-year survival.

## Abbreviations

APACHE II = Acute Physiology and Chronic Health Evaluation II; CI = confidence interval; CPC = cerebral performance category; CPR = cardiopulmonary resuscitation; ICU = intensive care unit; ILCOR = International Liaison Committee on Resuscitation; IQR = interquartile range; LOS = length of stay; MICU = medical intensive care unit; ROSC = return of spontaneous circulation.

## Competing interests

The authors declare that they have no competing interests.

## Authors' contributions

CS, IS, JCS, and DH designed and supervised the analysis and analyzed all data. MO was involved in the collection of all data and participated in the data analysis. AJ and AK participated in the design of the study, revised the manuscript for important intellectual content, and helped to draft the manuscript. CS and IS contributed equally to this work. All authors read and approved the final version of the manuscript.
